# Self-perception of cognitive sequels in post-COVID-19 individuals

**DOI:** 10.1590/1980-5764-DN-2022-0080

**Published:** 2023-05-05

**Authors:** Emily Viega Alves, Bárbara Costa Beber

**Affiliations:** 1 Universidade Federal de Ciências da Saúde de Porto Alegre, Porto Alegre RS, Brazil.

**Keywords:** Pandemics, COVID-19, Cognition, Neurologic Manifestations, Neuropsychology, Pandemias, COVID-19, Cognição, Manifestações Neurológicas, Neuropsicologia

## Abstract

COVID-19 is an infection, primarily respiratory, caused by the SARS-CoV-2, which can also affect the central nervous system, causing neuropsychological damage. There are studies describing post-COVID-19 cognitive deficits, but it is important to know this outcome in populations with different social, biological, and cultural characteristics.

**Objective:**

The aim of this study was to assess the self-perception of cognitive sequelae in post-COVID-19 individuals and identify whether there is a possible relationship between the outcome of the participants’ self-perception and sociodemographic and clinical data.

**Methods:**

This is a cross-sectional study, carried out through an online questionnaire on the Google Forms platform, in which sociodemographic data, general health data, clinical manifestations of COVID-19, and post-COVID-19 self-perception of the cognitive domains of memory, attention, language, and executive functions were collected.

**Results:**

The final sample consisted of 137 participants, and it was possible to identify that memory and attention were the domains with the highest impression of worsening post-COVID-19, followed by executive functions and language. In addition, it was identified that being female may be related to a worse self-perception of all cognitive functions and that having depression or other psychiatric diseases and obesity can significantly affect at least half of the cognitive domains evaluated.

**Conclusions:**

This study pointed to a post-COVID-19 cognitive worsening of the participants.

## RESUMO.

A COVID-19 é uma infecção, primariamente respiratória, causada pelo vírus SARS-CoV-2, mas que também pode atingir o sistema nervoso central, ocasionando danos neuropsicológicos. Há estudos que descrevem os déficits cognitivos pós-COVID-19, mas é importante conhecer esse desfecho em populações com diferentes características sociais, biológicas e culturais.

**Objetivo:**

Avaliar a autopercepção de sequelas cognitivas em indivíduos pós-COVID-19 e identificar se há uma possível relação entre o desfecho da autopercepção dos participantes e dados sociodemográficos e clínicos.

**Métodos:**

Trata-se de um estudo transversal, realizado com o uso de um questionário *online* na plataforma Google Forms, no qual foram identificados dados sociodemográficos, dados de saúde geral, manifestações clínicas da COVID-19 e a autopercepção dos domínios cognitivos de memória, atenção, linguagem e funções executivas pós-COVID-19.

**Resultados:**

A amostra final foi composta de 137 participantes, e pôde-se observar que memória e atenção foram os domínios com maior impressão de piora pós-COVID-19, seguidos por funções executivas e linguagem. Além disso, constatou-se que ser do gênero feminino pode estar relacionado com uma pior autopercepção de todas as funções cognitivas pós-COVID-19 e que ter depressão ou outras doenças psiquiátricas e obesidade pode afetar significativamente pelo menos metade dos domínios cognitivos avaliados.

**Conclusões:**

Este trabalho apontou para a piora cognitiva pós-COVID-19 dos participantes.

## INTRODUCTION

COVID-19 is an infection caused by SARS-CoV-2^1^. Due to its high transmission capacity^2^, including in the asymptomatic phases, COVID-19 remains a challenge in the health community worldwide^3^. It was declared a pandemic disease in March 2020^4^. Symptoms of this disease usually develop between the fifth and tenth day after exposure, and isolation is mainly recommended for those who have had contact with the virus for a long time. The most common manifestations include fever, cough, fatigue, and shortness of breath that can lead to subsequent pneumonia^5^.

Evidence suggests that in addition to these manifestations, the emergence of other symptoms and their permanence is a reality, impacting the quality of life and functionality of those who have had the disease. These sequels are known as post-COVID syndrome, or just long-term COVID, and develop from immunosuppressive to cardiac sequelae^6^. Moreover, research proposes that SARS-CoV-2 could be neuroinvasive, and its pathways to reach central nervous system (CNS) are different (through the olfactory nerve, blood circulation, ACE2 protein in the brainstem, immunological injury, neuronal pathways, enteric nervous system, and its sympathetic afferent neurons, among others)^7^.

Cases of COVID-19 associated with stroke, encephalopathy, encephalitis, meningitis, Guillain-Barré syndrome, and other neurological complications have been documented since the beginning of the pandemic^8^, and cognitive damage as a result of the possible injuries caused by these diseases is a common cause of complaints in neurological units^9^. Knowing that COVID-19 impacts the CNS and can cause complications and neurological diseases, it is relevant to think that pure cognitive impairments may be manifestations that remain once the respiratory syndrome is solved. Even in the absence of neurological diseases caused by COVID-19, studies have shown that cognitive deficits may occur in individuals who were infected^10^, affecting memory, attention, and executive function, particularly verbal fluency.

After the peaks of the pandemic have passed, the health systems now need to deal with the high demands arising from the long-term COVID-19. The UK National Health Service defined the post-COVID-19 syndrome as unexplained, different types of persistent signs or symptoms over 12 weeks, developed during or after the COVID-19 infection^11^. Our object of interest in this study is the cognitive symptoms. We believe that this population may need and seek assistance due to their cognitive complaints caused by COVID-19. Complaints reflect patients’ self-perceptions of their cognitive performance and may not correspond equally to their performance on cognitive formal tests. However, knowing how subjects perceive their cognition and how their self-perception is related to other sociodemographic and health characteristics is important to identify individuals who are more vulnerable and in need of health monitoring. Thus, the purpose of this study was to investigate the self-perception of cognitive sequelae in Brazilian individuals post COVID-19 and identify if there is a possible relationship between this outcome and sociodemographic and clinical data of the participants.

## METHODS

### Design and location

This is an exploratory cross-sectional study, with data collection online and registered at the Federal University of Health Sciences of Porto Alegre (UFCSPA).

### Participants

The sample was characterized as a convenience sample, composed of participants recruited from the Internet through the dissemination of the research on websites, social media, and the researchers’ email lists.

Inclusion criteria were as follows: participants aged 18 years or older, a declaration of previous COVID-19 infection confirmed by some test, and Internet access to answer the questionnaire. Participants who did not complete the questionnaire were excluded from the study.

### Data collection

Data collection took place through an online questionnaire organized into four sections on the Google Forms platform, which only allowed responses after participants read and confirm the informed consent form, which was available before the presentation of the questionnaire. Each section evaluated, respectively, sociodemographic data such as age, demographic area of Brazil, sex, race, and educational level; general health data such as participants’ self-perceptions of health and comorbidities; clinical manifestations of COVID-19, such as disease symptoms; and self-perception of post-COVID-19 cognitive domains. The questionnaire is available in Supplementary material. Since this research project generated a great amount of data, we selected only the variables of interest to reach the objectives of this specific study.

The cognitive domains measured, in terms of self-perception, were memory, language, attention, and executive functions. With the topic of memory, in section 4, the MAC-Q Subjective Perception of Memory Loss Questionnaire^12^ adapted for the purposes of this research was used. For the purpose of this study, we use only the question that asks a general description about memory. In the other domains, the questions were developed using the structure of the MAC-Q instrument as a model, due to the lack of validated self-perception questionnaires on these other domains in current literature. The questions were answered about cognitive perception in the present moment compared to pre-infection cognition through the following response options on a Likert scale: much better now, a little better now, no change, a little worse now, and much worse now. There was a definition of each cognitive domain in plain language prior to each question.

A pilot study was carried out, in which three volunteers answered the questionnaire and provided feedback to the researchers on the clarity of the instrument and its functioning. The problems pointed out were corrected by the researchers. The estimated average time to answer the questionnaire was approximately 10–15 min. To reach the purpose of this study specifically, only the answers to the questions that requested the general description of each cognitive domain were included in the analysis.

### Ethical aspects

This study was registered and approved by the ethics committee of the institution of origin under opinion number 4.970.521. The participants had contact with the description of the study and the informed consent form before the questionnaire was presented, which was released only after the individual had agreed to participate. For the development of this research, the requirements of the National Health Council, according to Resolution no. 466 of 2012, were adopted. Furthermore, this study followed all the recommendations of CIRCULAR OFFICE N 2/2021/CONEP/SECNS/MS, which deal with research in a virtual environment.

### Data analysis

Categorical variables were described as relative (%) and absolute (n) frequency, while continuous variables were described as mean and standard deviation. Data distribution was verified using the Shapiro-Wilk test. Spearman's test was used to investigate correlations between continuous variables. The Mann-Whitney U and Kruskal-Wallis tests were used to compare the scores of cognitive domains between responses of categorical variables, when there were two or three categories, respectively. For these analyses, the Statistical Package for the Social Sciences (SPSS) version 25 software was used.

The variables that presented p<0.20 were the candidates to be included in the multiple linear regression models. A violation of the assumption of non-autocorrelated errors has been seen (significant Durbin-Watson test). Therefore, the estimation method was changed to generalized least squares (GLS), weighted by 1/adjusted values², with a stepwise method of variable selection. Other assumptions were analyzed [normality of residuals, absence of multicollinearity (variance inflaction factor – VIF <5), and homoscedasticity (graphic inspection of residuals × predicted)]. For this analysis, the R software was used. The adopted significance level was 5%.

## RESULTS

### Sample description

A total of 148 individuals accessed the questionnaire. Of these, 1 was excluded for not agreeing with the informed consent and 10 for not answering the main outcomes of the study. Thus, 137 subjects made up the sample of the present study.

Table 1 sums up the participants’ sociodemographic and general health data.

**Table 1:** Description of the sociodemographic and health characteristics of the sample.

Variable		n (%) or mean (SD)
Age - mean (SD)		34.61 (15.04)
Gender (F) – n (%)		108 (78.8)
Race – n (%)	White	119 (86.9)
	Brown	12 (8.8)
	Black	6 (4.4)
Education in years – mean (SD)		15.16 (5.22)
Educational level – n (%)	Complete elementary education	1 (7)
	Incomplete elementary education	4 (2.9)
	Complete high school	10 (7.3)
	Incomplete high school	1 (7)
	Complete higher education	24 (17.5)
	Incomplete higher education	42 (30.7)
	Postgraduate	48 (35)
	Technical course	7 (5.1)
Region of Brazil – n (%)	South	119 (86.9)
	South east	13 (9.5)
	North	2 (1.5)
	North east	2 (1.5)
	Midwest	1 (0.7)
General self-perception of health – n (%)	Very bad	1 (0.7)
	Bad	3 (2.2)
	Reasonable	26 (19)
	Good	79 (57.7)
	Very good	28 (20.4)
Comorbidities – n (%)	Asthma	9 (7.1)
	Hypertension	16 (12.6)
	Depression and other psychiatric diseases	27 (21.3)
	Hypercholesterolemia	20 (15.7)
	COPD	4 (3.1)
	Obesity	11 (8.7)
	Diabetes	6 (4.7)
	Others	14 (11)
Number of symptoms – mean (SD)		7.54 (11.4)

Abbreviations: F: female; COPD: chronic obstructive pulmonary disease.

Table 2 describes the sample's self-perception of cognitive symptoms at the current time compared to pre-COVID-19 infection cognition.

**Table 2:** Description of the self-perception of cognitive domains.

Cognitive domain	Much worse now – n (%)	A little worse now – n (%)	No change – n (%)	A little better now – n (%)	Much better now – n (%)
Memory	17 (12.4)	74 (54)	46 (33.6)	–	–
Language	6 (4.4)	43 (31.4)	86 (62.8)	2 (1.5)	–
Attention	10 (7.4)	64 (47.1)	61 (44.9)	1 (0.7)	–
Executive functions	7 (5.1)	53 (39)	63 (46.3)	12 (8.8)	1 (0.7)

### Investigation of the association with sociodemographic aspects

Associations between sociodemographic aspects and general description scores in each cognitive domain were tested. There was a statistically significant difference with regard to sex, demonstrating that women had a worse perception in the general description of all cognitive domains (p<0.001 in all domains, Mann-Whitney U test). There was no significant association related to race (memory, p=0.289; language, p=0.234; attention, p=0.291; and executive functions, p=0.659; Mann-Whitney U test), age (memory, p=0.555; language, p=0.704; attention, p=0.143; and executive functions, p=0.669; Spearman's test), or education (memory, p=0.910; language, p=0.602; attention, p=0.659; and executive functions, p=0.160; Kruskal-Wallis test).

### Investigation of the association with clinical aspects

The association between the presence of pre-COVID-19 comorbidities and the self-perception of cognitive domains was tested, and the results are shown in Table 3. Table 4 explains the correlation found between the number of symptoms caused by the COVID-19 infection and the participants’ general self-perception of health, including cognitive self-perception.

**Table 3:** Association between pre-COVID-19 comorbidities and cognitive domains.

Comorbidity	Memory	Language	Attention	Executive functions
Asthma	0.448	0.020*	0.347	0.265
Obesity	0.019*	0.003*	0.326	0.112
Diabetes	0.597	0.195	0.313	0.392
Hypertension	0.012*	0.478	0.793	0.672
Hypercholesterolemia	0.014*	0.142	0.032*	0.002*
Depression or other psychiatric disease	0.042*	0.006*	0.002*	0.022*
COPD	0.029*	0.063*	0.114	0.013*
Others	0.436	0.968	0.520	0.962

Abbreviation: COPD: chronic obstructive pulmonary disease.

Notes:

* p≤0.05; Kruskal-Wallis test.

### Multiple linear regression analysis models

Multiple linear regression analysis models have been tested, including the univariate analysis models where the variables presented p<0.20 (described previously). These analyses have been performed in each cognitive domain, as described below:

- Memory: The variables that were the candidates for the multiple linear regression model were sex, medication use, hypertension, hypercholesterolemia, obesity, depression or other psychiatric illnesses, chronic obstructive pulmonary disease (COPD), number of COVID-19 symptoms, and general self-perception of health. The final model was composed of the variables presented in Table 5 and was able to explain 21.8% (adjusted R^2^ 0.218) of the variability in self-perception of memory.
- Language: The candidates for the multiple linear regression model were sex, race, obesity, depression or other psychiatric illnesses, COPD, diabetes, asthma, hypercholesterolemia, number of COVID-19 symptoms, and general self-perception of health. The final model was composed of the variables shown in Table 5 and was able to explain 30% of the variability in language self-perception (adjusted R^2^ 0.30).
- Attention: Sex, COPD, depression or other psychiatric illnesses, general self-perception of health, and hypercholesterolemia were the candidates for the multiple linear regression model in this domain. The final model explained 10.3% of the variability in participants’ self-perception of attention ability (Table 5) (adjusted R^2^ 0.103).
- Executive functions: Sex, medication use, obesity, depression or other psychiatric illnesses, COPD, general self-perception of health, and hypercholesterolemia were the candidates for this regression model. The final model explained 17.5% of the variability in self-perception of executive functions (adjusted R^2^ 0.175).

**Table 4:** Correlation between the number of COVID-19 symptoms during infection, general self-perception of health, and cognitive sequelae.

Cognitive domain	Number of symptoms COVID-19 – r (p)	General self-perception of health- r(p)
Memory	-0.201 (0.019)*	0.243 (0.004)*
Language	-0.188 (0.029)*	0.178 (0.038)*
Attention	-0.020 (0.820)	0.163 (0.058)
Executive functions	-0.052 (0.547)	0.274 (0.001)*

Notes:

* p≤0.05; Spearman's test.

**Table 5:** Multiple linear regression models of the investigated cognitive domains.

Variable included in the final model		β	Standard error	t	p-value
Memory	(constant)	2.23	0.27	8.20	<0.001
	General self-perception of health	0.19	0.06	3.18	0.002
	Female	-0.40	0.11	-3.79	<0.001
	Number of COVID-19 symptoms	-0.04	0.02	-2.35	0.021
	Obesity	-0.34	0.16	-2.17	0.032
Language	(constant)	3.07	0.11	28.47	<0.001
	Obesity	-0.49	0.12	-4.14	<0.001
	Asthma	-0.50	0.13	-3.86	<0.001
	Depression or other psychiatric disorders	-0.24	0.08	-2.95	0.004
	Female	-0.21	0.08	-2.44	0.016
	Number of COVID-19 symptoms	-0.03	0.01	-2.05	0.043
Attention	(constant)	2.67	0.11	24.11	<0.001
	Depression or other psychiatric disorders	-0.37	0.12	-3.15	0.002
	Female	-0.28	0.12	-2.32	0.022
Executive functions	(constant)	2.16	0.28	7.74	<0.001
	General self-perception of health	0.20	0.07	3.01	0.003
	Female	-0.29	0.11	-2.56	0.012
	COPD	-0.64	0.28	-2.29	0.024

Abbreviations: COPD: chronic obstructive pulmonary disease.

## DISCUSSION

COVID-19 can be a threat in several areas of the human body, with some evidence suggesting that it can cause damage to cognitive domains, as it has neuroinvasive mechanisms^7^. In the present study, we sought to assess, through the assessment of self-perception, the possible cognitive sequelae of COVID-19 and identify relationships between this outcome and the sociodemographic and clinical data of the participants. It is of great scientific and clinical relevance to describe these symptoms, especially with regard to the study of different ways to prevent possible future harm related to the infection and guide patients on strategies to minimize the impact of the damage caused. Although post-COVID-19 cognition has already been tested in other studies^10^ and even evaluated through its self-perception for some domains^13^, we are not aware of studies that had as their main objective to investigate the cognitive self-perception including all cognitive domains of memory, attention, executive functions, and language in a standardized way.

In this study, it was possible to identify that memory and attention were the domains with the highest impression of worsening post-COVID-19, followed by executive functions and language, which also presented a percentage higher than 35% of participants who have perceived worsening. These findings corroborate other studies present in the literature^9^ that investigated neuropsychological issues related to COVID-19.

In general, findings in the literature point to a higher incidence of post-COVID-19 memory symptoms. In Brazil, a group of researchers studied the cognitive effects of the disease in more than 400 patients who underwent a battery of neuropsychological examinations and tests after hospitalization. In this research, reports of memory loss exceeded 50% of participants^14^. In the case of the present study, memory also had the worst self-perception among the participants, reaching over 65% of “a little worse now” and “much worse now” answers.

A German study^15^ highlighted “attention and concentration” as the domain most significantly affected, even by those who had COVID-19 in its mildest form, suggesting that regardless of the degree of infection, cognitive symptoms can be present and harmful. In our work, attention was also perceived as altered by the participants and showed no association with the number of COVID-19 symptoms, which can be considered a measure of disease severity.

Social isolation due to the pandemic has been highlighted as a consequence that impacts executive functions^16^. However, few studies relate the influence of COVID-19 to this domain. An Italian study^17^ found that in the teenage population, getting COVID-19 and being hospitalized had an impact on the executive functions of individuals, especially with regard to working memory. In the present study, executive functions were also widely reported as modified. However, an important point to be taken into account is that 8.8% of the sample reported that their executive functions were a little better after COVID-19, and 0.7% said that they were much better now. These numbers may be related to the way in which the participants perceive themselves after an event that may have been considered traumatic for them. This is apparent since the questions in the questionnaire talk about planning capacity, decision-making, and changing strategies, and this may be more related to defense behaviors acquired by post-COVID individuals.

Finally, the cognitive domain that was most marked as “no changes” was language. However, more than 30% of the participants reported some type of difficulty after this period. This work is the first to study possible damage to language function from the point of view of the individuals, since linguistic impairments caused by neurological disorders resulting from infection, such as aphasia due to post-COVID-19 stroke, are more frequently reported^18^.

In addition to verifying the frequency of participants’ self-perception of cognition after COVID-19 infection, multiple linear regression models were used to investigate which sets of variables could explain the variability in participants’ self-perception performance in each cognitive domain studied. Among the variables that composed the final models, attention is drawn to the variable “female gender,” which was present in all final models. This result indicates that women presented a worse self-perception of all the investigated cognitive functions. The literature points out that a potential factor that affects self-perception of health is gender, since socially constructed roles and functions accommodate greater morbidity risks for women^19^. The Pan American Health Organization and the Economic Commission for Latin America and the Caribbean have disclosed the devastating impacts of COVID-19 on women^20^, both in social and economic matters, which can affect their general health, resulting in cognitive comorbidities.

Regarding the relationship between pre-COVID-19 comorbidities and self-perception of cognition of the participants, “depression or other psychiatric illnesses” was a predictor of worse perception of language and attention post-COVID-19. It is known so far that psychological and psychiatric disorders are risk factors for infection, hospitalization, and death from COVID-19^21^, because they are the potential facilitators in the process of exposure to SARS-CoV-2 and interfere with behavior patterns that healthcare providers need to be aware of. Furthermore, in our vision, self-perception of loss of functionality due to cognitive symptoms developed post-COVID-19 could also act as a trigger for depressive symptoms.

In our study, obesity was a predictor of worse language and memory perception, while COPD was a predictor of executive functions and asthma of language. Despite the absence of a clear relationship between those comorbidities and cognition in individuals with COVID-19, there are studies showing some associations between those comorbidities without COVID-19 and neuropsychological deficits^22–26^. Then, we believe that conditions such as obesity, COPD, and asthma could make cognitive functioning more vulnerable to the neurological dysfunction caused by COVID-19. In the case of asthma, it is also possible that the participants had in mind aspects related to speech and phonation, which are directly dependent on their breathing capacity, which can be more compromised among asthmatics. A study that sought to examine laryngeal function in patients with severe asthma found that 87% of their sample had laryngeal dysfunction affecting breathing, phonation, or both^27^.

In this study, we investigated the association of cognition with the number of COVID-19 symptoms, as an indirect measure of infection severity. This variable was a predictor of worse self-perception of memory and language. To date, the literature does not have concrete evidence that the number of symptoms is associated with cognitive deterioration. Nevertheless, some studies suggest that post-COVID-19 neuropsychological and neuropsychiatric sequelae tend to be more evident in more severe cases of the disease, especially with symptoms of memory loss^28^.

The subjects’ perception of general health was also assessed and has entered the final models of the executive functions and memory domains. Health self-perception investigates various aspects of physical health, functional capacity, and cognition^29^. The study by Freitas et al. was the first Brazilian study to relate self-perception of general health with cognition in the elderly population, showing that negative self-perception of health is related to greater cognitive decline^30^.

The present work had limitations that should be taken into account when interpreting the results and should be considered in future studies. First, the variables of educational level of participants and region of Brazil where they came from can be considered a sampling bias, especially due to the scope of dissemination of the study by the researchers, as well as the predominantly female, white, and young population. Furthermore, the lack of data collection on vaccinated and unvaccinated participants precluded a possible evaluation scenario on vaccines and their representativeness in cognitive issues related to COVID-19. We also suggest that future studies investigate the impact of post-COVID on labor life and how individuals perceive that impact.

This study pointed to a perceived cognitive worsening among the post-COVID-19 participants, especially in the domains of memory and attention. The sociodemographic variable that was most present in explaining the variability in the self-perceptive performance of individuals in each cognitive domain was the female gender, and among the comorbidities of all participants were depression and other psychiatric diseases as well as obesity.
